# De novo assembly of plant body plan: a step ahead of Deadpool

**DOI:** 10.1002/reg2.68

**Published:** 2016-10-28

**Authors:** Abdul Kareem, Dhanya Radhakrishnan, Yash Sondhi, Mohammed Aiyaz, Merin V. Roy, Kaoru Sugimoto, Kalika Prasad

**Affiliations:** ^1^ School of BiologyIndian Institute of Science Education and ResearchThiruvananthapuramKerala695016India; ^2^ Department of Applied Biological ScienceFaculty of Science and TechnologyTokyo University of Science2641 YamazakiNodaChiba278‐8510Japan

**Keywords:** auxin, callus, cytokinin, de novo organogenesis, pluripotency, regeneration, stem cells, transdifferentiation

## Abstract

While in the movie Deadpool it is possible for a human to recreate an arm from scratch, in reality plants can even surpass that. Not only can they regenerate lost parts, but also the whole plant body can be reborn from a few existing cells. Despite the decades old realization that plant cells possess the ability to regenerate a complete shoot and root system, it is only now that the underlying mechanisms are being unraveled. De novo plant regeneration involves the initiation of regenerative mass, acquisition of the pluripotent state, reconstitution of stem cells and assembly of regulatory interactions. Recent studies have furthered our understanding on the making of a complete plant system in the absence of embryonic positional cues. We review the recent studies probing the molecular mechanisms of de novo plant regeneration in response to external inductive cues and our current knowledge of direct reprogramming of root to shoot and vice versa. We further discuss how de novo regeneration can be exploited to meet the demands of green culture industries and to serve as a general model to address the fundamental questions of regeneration across the plant kingdom.

## 1 INTRODUCTION

Wear and tear is an inevitable part of normal growth and development in all lifeforms. An organism which cannot repair wounds and regenerate lost body parts will have compromised survival fitness compared to its rivals. Across kingdoms, nature has equipped living cells and tissues of organisms with a remarkable capacity to regenerate in order to conquer biotic and abiotic threats. Both plants and animals share this capacity as a common means of tissue repair despite having independent evolutionary origins (Birnbaum & Sánchez Alvarado, 2008; Sugimoto, Gordon, & Meyerowitz, 2011; Pulianmackal, Kareem, Durgaprasad, Trivedi, & Prasad, 2014). The underlying mechanism of regeneration is likely to have adopted similar strategies in both the kingdoms during evolution in concurrence with other developmental processes (Meyerowitz, 2002). The last decade has witnessed an extensive interrogation into regeneration in plants and animals in an attempt to unearth the molecular mechanism and to draw parallels between the two kingdoms (Takahashi & Yamanaka, 2006; Xu et al., 2006; Gordon et al., 2007; Birnbaum & Sánchez Alvarado, 2008; Sena, Wang, Liu, Hofhuis, & Birnbaum, 2009; Sugimoto, Jiao, & Meyerowitz, 2010; Sanchez Alvarado & Yamanaka, 2014; Kareem et al., 2015; Efroni et al., 2016; Ikeuchi, Ogawa, Iwase, & Sugimoto, 2016). Regeneration has been a focus of attention to study the basic principles of cellular plasticity and other developmental processes. In addition, this knowledge has been exploited in regenerative medicine and horticulture.

Plants have the remarkable ability to regenerate an entire plant from a few existing cells and also to regrow lost parts. The regeneration potential of plants has been recognized for long but it is a more recent discovery in animals (Birnbaum & Sánchez Alvarado, 2008). Regeneration has been observed in plants ranging from lower forms like algae and bryophytes to higher flowering plants (Birnbaum & Sánchez Alvarado, 2008; Duclercq, Sangwan‐Norreel, Catterou, & Sangwan, 2011; Pulianmackal et al., 2014; Ikeuchi et al., 2016). Bud formation upon the decortication of elm tree demonstrated by Duhamel in 1756 is considered to be the pioneering attempt in plant regeneration (Gautheret, 1985). Experimental approaches to mimic this natural capacity under in vitro culture conditions became feasible after realizing the totipotent nature of plant cells (Haberlandt, 1902). Thanks to the work of Skoog and Miller on the identification of suitable hormonal combinations (Skoog & Miller, 1957), in vitro formation of entire plants or organs is now a simple process which has been widely exploited in various plant species for the past 60 years (Smith, 2013; Vasil & Thorpe, 2013). However, unlike in plants, de novo organogenesis under simple hormonal combinations is yet to be a reality in animals.

**Figure 1:**
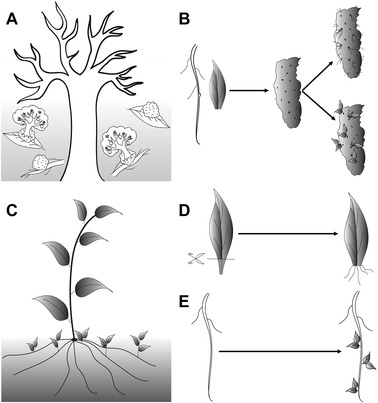
Cartoon showing various modes of regeneration in plants. (A) Regeneration potential of various plant organs. (B) Indirect regeneration of root and shoot from callus on suitable inductive medium. (C) Natural regeneration of shoot from root. (D) Direct regeneration of root from wounded leaf explants on hormone‐free medium. (E) Direct shoot regeneration from root explants on suitable inductive medium

De novo organogenesis in plants can be achieved either directly or indirectly (Fig. 1); however, cellular reprogramming is inevitable in both the modes. During direct regeneration, the cells undergo transdifferentiation wherein root cells are reprogrammed to shoot and vice versa (Chatfield et al., 2013; Liu et al., 2014; Kareem et al., 2016). The indirect mode involves the formation of an intermediate regenerative mass of cells called callus from adult stem cells that are distributed throughout the plant body (Sugimoto et al., 2010, 2011; Duclercq, Sangwan‐Norreel, Catterou, & Sangwan, 2011). The callus in turn produces root or shoot in response to an appropriate ratio of the key plant hormones auxin and cytokinin (Valvekens, Van Montagu, & Van Lijsebettens, 1988; Gordon et al., 2007; Atta et al., 2009). Nevertheless, the primary step towards organ regeneration is the acquisition of competence. Once cells have acquired the competence, the dynamic assembly of molecular interactions defines the patterning of new meristem and organ primordia. In this review we discuss de novo regeneration in plants in response to external inductive cues and its plausible comparison with animal counterparts. We focus on cell‐type specificity, regulatory modules conferring the competence and the assembly of regulatory interactions leading to the completion of regeneration.

## 2 NATURE OF THE EXPLANT AND DE NOVO REGENERATION

As per cell theory, new cells arise from pre‐existing cells (Virchow, 1859). In plant regeneration, the source of all new tissues is the explant procured from the mother plant which is to be propagated. The clonal nature of in vitro culture ensures that both beneficial and disadvantageous characters of the donor plant are transmitted to the newly generated plants. Therefore, the right choice of explant is the first and most important step in plant regeneration. An explant denotes any part of the parental plant such as embryo, root, hypocotyl, cotyledon, parts of shoot like shoot tip, leaf, petiole, node, internode, inflorescence and parts of flower (Halperin, 1986; Valvekens et al., 1988; Weigel & Glazebrook, 2002; George, Hall, & De Klerk, 2007; Sugimoto et al., 2010). Factors such as availability, age of explants, response rate, contamination susceptibility, lethality of phenolic exudations, and the objective of tissue culture govern the suitability of the explant (Smith, 2013).

In vitro plant regeneration may be performed for a variety of purposes and each requires the most suitable explant type. For the general purpose of micropropagation, any explant that possesses the maximum regeneration efficiency can be used. However, the generation of haploid plants requires the use of haploid explants such as microspores, egg cells, or tissues bearing the same, such as anther, ovary, or inflorescence (George et al., 2007; Vasil & Thorpe, 2013). A commonly used explant for hairy root induction by *Agrobacterium rhizogenes* for secondary metabolite production is root tip (Flores, Hoy, & Pickard, 1987). To generate virus‐free plants, shoot apical meristem is the best choice of explant due to the meristematic nature and the lack of connection to differentiated vascular tissue which prevents the spread of viral infection (Slack & Tufford, 1995). The problem of endophytic microbial associations can be overcome by the use of tissues from plants grown in vitro. This can also help in the conservation of the natural population of the donor plant.

Despite the availability of a wide range of explants, the regeneration response relies heavily on the nature of the explant. The response varies between species, genotypes, ecotypes, organs of the same plant, and even between sections of the same organ (Coleman & Ernst, 1989; Akama et al., 1992; Siemens, Torres, Morgner, & Sacristán, 1993; Zhang, Takahata & Xu, 1998; Motte et al., 2014). In *Arabidopsis*, root and hypocotyl have been shown to be the explants with the highest regeneration potential while cotyledon displays the lowest regeneration potential (Valvekens et al., 1988; Akama et al., 1992). The underlying cause of differences in regeneration potential between various explants may be the presence of more regenerative cells in the explant tissue of higher efficiency. Unlike in *Arabidopsis*, cotyledon displays the highest shoot regeneration potential in *Brassica* spp. (Tang et al., 2003; Guo, Zhu, Hu, & Zheng, 2005). This implies that the regeneration potential of the same tissue can vary in different species. In addition, the extrinsic cues such as hormones and culture conditions required for organogenesis may vary for diverse explants (Sugimoto et al., 2010). The endogenous cues from the donor plant to which the explant has been habituated may also have a role in in vitro response. For instance, leaf explants closer to the shoot apex are more responsive in culture (Chaudhuri, Pal, & Jha, 2008). This enhanced response may be due to the relatively young developmental stage of the explants closer to the shoot apex.

Age of the explant is an important factor that influences regeneration capacity (Sugimoto & Meyerowitz, 2013). It has been observed that older leaf explants have reduced root and shoot regeneration efficiency compared to younger leaf explants (Chen et al., 2014; Zhang et al., 2015). The reduced regeneration of root and shoot is partly attributed to the reduced levels of free endogenous auxin and defective cytokinin signaling mediated by micro RNA (miR156), respectively. In older explants there is a decline in miR156. As a result SQUAMOSA PROMOTER BINDING PROTEIN LIKE (SPL), which is normally under the repression of miR156, interferes with the transcriptional activity of B‐type *ARABIDOPSIS RESPONSE REGULATORS* (*ARR*s) and impedes the cytokinin signaling pathway. Thus, the shoot regeneration capacity is adversely effected (Zhang et al., 2015). Providing the required hormone extrinsically in the culture medium can recover the regeneration potential of the older explant to some extent under these conditions. Another hormone that is influenced by the aging of the plant is abscisic acid (ABA). ABA in combination with cytokinin enhances shoot regeneration (Paulraj & Yeung, 2012). However, the underlying molecular mechanism is yet to be studied. Age dependence may also be a contributing factor to the loss of plasticity and competence to proliferate and the inability to switch fates in more mature tissues compared to the totipotent embryonic status of younger plant cells.

Regardless of the effort of standardizing the above mentioned factors, the response of a different genotype or ecotype of a plant may still show variation in regeneration efficiency (Motte et al., 2014). The regeneration potential of root explant varies from 0% to 100% in various *Arabidopsis* accessions. Among the commonly used laboratory ecotypes of *Arabidopsis*, Wassilewskija (Ws) shows higher regeneration potential than the other popular ecotypes Columbia (Col‐0) and Landsberg *erecta* (L*er*) (Chaudhury & Signer, 1989; Akama et al., 1992; Motte et al., 2014). The basic cause of this difference in regeneration capacity is due to the genetic differences that may have arisen as a part of inbreeding and crossbreeding in the case of genotype and as a result of prolonged growth and adaptations to a particular environmental condition in the case of ecotypes. The key quantitative trait loci involved in this variation have been characterized by combined linkage and association mapping (Motte et al., 2014).

As the saying goes, well begun is half done. Therefore, the wise choice of the explant is a prerequisite to obtain desired in vitro response with maximum regeneration capacity. This will require standardization of a combination of culture parameters, keeping in mind the dynamics of the endogenous growth hormones and the physiological status of the explant.

## 3 CELL‐TYPE SPECIFICITY, CONCEPT OF ADULT STEM CELLS AND FORMATION OF REGENERATIVE MASS

**Figure 2:**
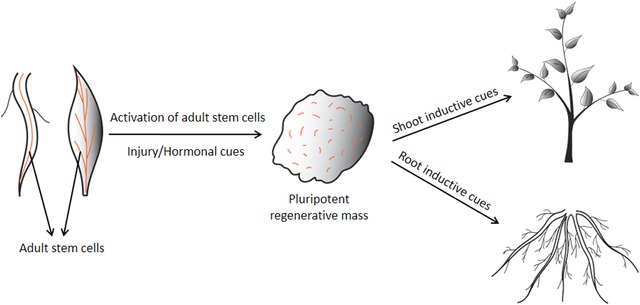
Schematic representation of the two stages involved in callus mediated de novo regeneration

When explants are exposed to the phytohormone auxin present in callus inducing medium, a proliferating regenerative callus forms. The regenerative callus can further give rise to either root or shoot depending upon the external inductive stimulus. This is attributed to plant cell totipotency which confers the ability to elaborate an entire plant body. For long, it had been thought that all plant cells are totipotent and can undergo dedifferentiation to generate callus (Birnbaum & Sánchez Alvarado, 2008). But recent developments in the understanding about cell‐type specificity and differentiation status during callus formation have redefined the concepts of cellular potency and reprogramming (Sugimoto et al., 2010, 2011; Ikeuchi, Sugimoto, & Iwase, 2013). Unlike previously thought, not all somatic cells can form callus. It is now evident that callus formation is initiated by the activation of a specialized population of partially differentiated cells which span throughout the plant body (Fig. 2). These partially differentiated cells are called adult stem cells (Sugimoto et al., 2011). In general, adult stem cells are defined as pre‐existing stem cells that can proliferate and differentiate into various cell types upon appropriate inductive cues. The adult stem cell populations in plants comprise pericycle and procambium cells (Sugimoto et al., 2010, 2011; Liu et al., 2014) which have been known to generate post‐embryonic structures such as lateral root and vascular tissue respectively (Mähönen et al., 2000; De Smet, Vanneste, Inze, & Beeckman, 2006). However, the concept of adult stem cells has to be better defined in plants as in animals (Sugimoto et al., 2011; Rezza, Sennett, & Rendl, 2014).

Several studies support the highly versatile nature of pericycle cells which empower them as adult stem cells. In a post‐embryonic root, pericycle cells remain quiescent all along the root except at the sites opposite to the xylem poles from where lateral root initiates (Malamy & Benfey, 1997; De Smet et al., 2006). Under specific external inductive cues the xylem pole pericycle cells can also give rise to callus or convert into shoot fate (Atta et al., 2009; Sugimoto et al., 2010; Kareem et al., 2015, 2016). The flexible nature of pericycle cells is explained by their partial differentiation status, but the molecular basis for the partial differentiation status is unknown. However, some studies have shown that non‐pericycle cells can also give rise to callus. During the protoplast culture of *Arabidopsis* leaf after the enzymatic removal of cell wall, mesophyll cells can be reprogrammed into the callus fate (Chupeau et al, 2013). Similar observations have been reported in several *Nicotiana* spp. (Bourgin, Chupeau, & Missonier, 1979) and also in green algae *Bryopsis plumose* (Kim, Klotchkova, & Kang, 2001). Moreover, mutants defective in biosynthesis of cell wall components such as pectin and cellulose show hormone‐independent callus formation (Frank et al., 2002; Iwai, Masaoka, Ishii, & Satoh, 2002). This opens up the prospect of reprogramming additional cell types for callus induction. The removal of cell wall is likely to impact the state of cells by altering the mechanical properties such as turgor pressure and the stress experienced by cells thereby triggering callus formation.

It is interesting to examine if cellular reprogramming during pluripotent callus formation will lead to embryonic ground state. Callus displays a gene expression pattern resembling that of the basal half of the embryo. From this, one may infer that callus has basal embryo‐like features. But several lines of evidence suggest that the formation of pluripotent callus largely follows the molecular developmental program of lateral root initiation (Sugimoto et al., 2010). The strongest evidence is that *aberrant lateral root formation 4* (*alf4*) mutant which fails to form lateral root (Celenza, Grisafi, & Fink, 1995) also does not form callus (Sugimoto et al., 2010). Similarly *plethora3* (*plt3*)*;plt5;plt7* triple mutant makes lateral root primordia (LRP) but these cells are not pluripotent as they fail to develop all cell types of lateral root (Prasad et al., 2011; Hofhuis et al., 2013). This mutant is able to make callus but it is not pluripotent as it fails to regenerate organs (Kareem et al., 2015). But neither *alf4* nor *plt3;plt5;plt7* mutants show any defects in embryogenesis (Celenza et al., 1995; Prasad et al., 2011). Therefore, callus formation from various parts of plants follows a lateral root development pathway and callus predominantly displays a gene expression pattern similar to lateral root meristem (Sugimoto et al., 2010; Kareem et al., 2015). These studies suggest that callus may not be an undifferentiated tissue; rather it possesses differentiated root‐like features. Further, callus does not go back to the zero point of embryonic state to achieve the pluripotent state. An intriguing question arises why callus derived from various organs should retain root‐specific genes. Insights into the functional significance of root‐specific genes in the callus emerge from recent studies which show that calluses which fail to express root stem cell maintenance regulators are not pluripotent (Kareem et al., 2015).

## 4 GENETICS UNDERLYING CALLUS FORMATION

### 4.1 Pericycle competence

The formation of callus is under the tight control of the molecular machinery which determines cellular competence to re‐enter the cell cycle. One of the important factors that provides competence to pericycle cells is *ALF4* whose loss of function mutant is defective in callus formation from multiple organs such as root, cotyledon, and petal (Sugimoto et al., 2010). ALF4 is a transcription factor expressed in multiple organs to maintain the cells in a mitotically competent state. Initially *alf4* mutant has been described to be deficient in LRP formation due to the absence of cell division in pericycle cells (Celenza et al., 1995). These studies provide strong evidence for the direct link between the necessity of pericycle competence for the formation of LRP and callus (Sugimoto et al., 2010). The necessity of *ALF4* for the division of leaf protoplast suggests its key role in making non‐pericycle cells also competent to enter the regeneration program (Chupeau et al., 2013). In addition, *ALF4* is required for the wound healing process during grafting (Melnyk, Schuster, Leyser, & Meyerowitz, 2015). A recent study demonstrates that the cell layer signal of very‐long‐chain fatty acids (VLCFAs) restricts the callus forming capacity of pericycle cells in *Arabidopsis* partly by inhibiting *ALF4* transcription (Shang et al., 2016). Deficiency in VLCFAs leads to enhanced callus forming capacity of pericycle cells by elevating the *ALF4* transcription. Thus VLCFA mediated *ALF4* signaling acts as a stringent control to prevent excess callus formation from pericycle upon external hormone application. It also suggests how the differentiated state of a cell is maintained during normal plant growth and development. It is interesting to note that the VLCFA mediated pericycle competence for callus formation can be uncoupled from the *SOLITARY ROOT* (*SLR*) mediated lateral root initiation. The *slr* mutant is defective in both lateral root formation and callus initiation. The ectopic expression of *ALF4* in *slr* background can only rescue the callus formation but not the lateral root development (Fukaki, Tameda, Masuda, & Tasaka, 2002; Shang et al., 2016). These studies imply that the developmental programs leading to callus formation might not be sufficient to trigger lateral root formation.

### 4.2 Hormonal signaling cascade and induction of key regulators

While VLCFA regulated *ALF4* is the initial trigger, auxin‐rich culture medium induces a cascade of molecular players. For instance, auxin activates four LATERAL ORGAN BOUNDARIES DOMAIN (LBD) transcription factors that act downstream to *AUXIN RESPONSE FACTOR 7* (*ARF7*) and *ARF19* to initiate callus formation (Fan, Xu, Xu, & Hu, 2012). The ectopic overexpression of these transcription factors, namely LBD16, LBD17, LBD18, and LBD29, can trigger callus formation without the supply of exogenous auxin. In contrast, suppression of these transcription factors inhibits callus formation even in the presence of auxin, suggesting the key role of these genes in callus formation (Fan et al., 2012). The functional significance of both *ARF* and *LBD* genes in lateral root formation has been identified (Okushima, Fukaki, Onoda, Theologis, & Tasaka, 2007) which emphasizes the notion that callus formation largely follows a lateral root development program. The downstream pathway of *LBD* mediated callus formation is vague. However, based on studies on *LBD* mediated lateral root development the downstream mechanism of callus formation can be inferred. During lateral root development, *LBD18* activates cell wall loosening factor EXPANSIN (Lee & Kim, 2013; Lee, Kim, Kim, Lee, & Kim, 2013). Together with *LBD33, LBD18* induces the cell cycle regulator E2 PROMOTER BINDING FACTOR a (E2Fa) which dimerizes with DIMERIZATION PARTNER (DP) protein for cell cycle entry (Berckmans et al., 2011). However, these cell cycle regulators alone are insufficient to trigger callus compared to *LBD* genes (Ikeuchi et al., 2013). Additional cell cycle regulators induced by *LBD* and other transcription factors need to be identified to further our understanding of the key transcription factor mediated mechanism of callus formation. Nevertheless, cell cycle activation is a key regulatory checkpoint during callus formation.

A recent study has shown that callus formation is also regulated by miRNA mediated interplay between auxin and cytokinin signaling (Liu et al., 2016). *miR160* acts as a key repressor of callus formation by cleaving the mRNA of auxin signaling gene *ARF10* and subsequently activating cytokinin signaling gene *ARR15*. Thus endogenous hormonal signaling interactions play a critical role in callus formation despite the external hormone supplement.

### 4.3 Establishment of pluripotent state

The sheer formation of callus may not determine the ability to regenerate. Callus needs to be pluripotent for the subsequent regeneration of different organs such as shoot and root. The pluripotent state of callus is established by root stem cell maintenance regulators (Kareem et al., 2015) such as AP2/ERF transcription factors PLT1 and PLT2 (Aida et al., 2004; Mähönen et al., 2014). The three redundantly acting transcription factors PLT3, PLT5, and PLT7 (Nole‐Wilson, Tranby, & Krizek, 2005; Prasad et al., 2011) regulate the activity of the root stem cell maintenance regulators PLT1 and PLT2 to establish pluripotency in the callus derived from various organs such as leaf, cotyledon, hypocotyl, and root (Kareem et al., 2015). *PLT3*, *PLT5*, and *PLT7* are rapidly induced upon external auxin application. The cumulative loss of function of these genes (*plt3;plt5;plt7*) leads to the formation of callus lacking the expression of root stem cell maintenance regulators despite the auxin‐rich culture medium. The mutant callus completely loses the potential to regenerate and the reconstitution of root stem cell maintenance regulators re‐establishes the pluripotent state. In addition to PLT1 and PLT2, other root‐specific stem cell maintenance regulators are also likely to function in a similar fashion under the control of PLT3, PLT5, and PLT7. Root stem cell maintenance regulators are predominantly expressed in callus during its induction phase and reinstate the pluripotent state. These are downregulated when the callus is shifted to cytokinin medium for shoot induction. When the high level is sustained even upon shoot induction by forced expression in a heterologous fashion, a default pathway of root formation is opted for despite the abundance of cytokinin. This signifies the tight temporal and spatial control of root stem cell maintenance regulators during regeneration. The necessity of root stem cell maintenance regulators to establish the pluripotent state in callus emphasizes the significance of the root‐like trait of callus derived from any part of the plant (Sugimoto et al., 2010; Kareem et al., 2015). Intriguingly, some of the mutant combination of root stem cell maintenance regulators such as *PLT2* and *PLT4* are embryonic lethal (the mutant cannot make all the embryo cell types) (Galinha et al., 2007). This suggests that, in all the scenarios such as embryogenesis, lateral root formation, and callus formation, the common function of root stem cell maintenance regulators is to establish the pluripotent state. Future studies are required to understand how root stem cell regulators establish the pluripotency.

### 4.4 Wound induced callus formation

In addition to auxin induced callus formation, pluripotent callus can also be formed through other pathways. Wound induced callus formation is one such pathway (Iwase et al., 2011, 2015). Wound induced callus formation is regulated by AP2/ERF transcription factor WOUND INDUCED DEDIFFERENTIATION 1 (WIND1). Ectopic induction of *WIND1* causes hormone‐independent callus formation from epidermal cells of root, hypocotyl, and cotyledon (Iwase et al., 2011). However, it is not clear how wounding induces *WIND1* expression and further how *WIND1* activates callus formation. Interestingly many of the genes that are expressed in auxin induced callus are also expressed in wound induced callus. So it is likely that the *WIND1* regulated pathway utilizes known auxin induced regulators of callus formation. *WIND1* is shown to activate the cytokinin signaling pathway. Previously, there have been reports that genes that are activated by auxin are also induced by cytokinin, for example *PLT*s (Hofhuis et al., 2013; Kareem et al., 2015). Therefore, it is possible that *WIND1* may work in activating genes that are also activated by auxin such as *PLT*s. A detailed analysis of *WIND1* mediated callus formation can provide deeper insights into the wound induced signaling pathway.

### 4.5 Epigenetics of callus formation: discriminating the tissue specificity

Besides genetic regulators, callus formation is also under the control of epigenetic regulators. Epigenetic changes involve genome‐wide changes in DNA methylation and histone modifications (Ikeuchi et al., 2015). Recent studies have shown that tissue‐specific callus formation is regulated by several epigenetic factors. For instance, epigenetic regulators belonging to Polycomb Repressive Complex 2 (PRC2) play a pivotal role in callus formation from leaf explants by regulating histone H3 lysine 27 trimethylation (H3K27me3) (He, Chen, Huang, & Xu, 2012). Differential epigenetic regulation partly explains the basis for the distinction between callus forming ability of leaf and root. Leaf explants but not the root explants of *curly leaf; swinger* (*clf; swn*) double mutants and *embryonic flower 2* (*emf‐*2) are defective in callus formation. Questions remain to be answered whether such differences are because additional leaf‐specific cells other than pericycle‐like cells (adult stem cells) get incorporated in callus formation. Also it needs to be determined whether the leaf‐specific cells need to lose their identity by epigenetic remodeling before they behave similarly to adult stem cells or pericycle‐like cells in a distinct organ context. Auxin induced transcription factor mediated changes in the cell fate can lead to an altered epigenetic landscape of cells. Moreover, transcription factors can recruit epigenetic regulators to the target loci (Weiste & Droge‐Laser, 2014). Thus callus formation is a result of the collective efforts of both genetic and epigenetic regulators.

Taken together callus formation which is an integral phase of indirect regeneration is regulated by a multitude of genetic and epigenetic factors. So far, only a few such players have been characterized. Further studies are required to understand the crosstalk between the various known molecular players, such as ALF4, LBDs, and PLTs, that regulate the formation of pluripotent callus. The discovery of newer factors and investigations of regulatory interactions between them should generate a larger regulatory network controlling callus formation.

## 5 PLAUSIBLE COUNTERPART OF CALLUS IN OTHER ORGANISMS

The formation of blastema in animals can be considered analogous to callus formation. Blastema formation is one of the first steps in epimorphic regeneration predominantly seen in certain lower animals (McLean & Vickaryous, 2011). Appendage loss causes cells that are undifferentiated, pluripotent or multipotent, from various origins to gather at the wound site to form a regenerating blastema. Thereafter, extensive proliferation occurs and the cells slowly re‐differentiate into the cells required to form tissue for regrowing the lost appendage (Christen, Robles, Raya, Paramonov, & Izpisua Belmonte, 2010). More recently, it has been demonstrated that mature adult cells can be reprogrammed to resemble embryonic stem cells using a combination of chemical and genetic cues (transcription factors). These cells, called induced pluripotent stem cells (iPSCs), have the capacity to develop into any kind of cells in the body (Takahashi & Yamanaka, 2006; Takahashi et al., 2007; Lin & Wu, 2015). Callus and iPSCs are similar in their potency, that is, both cells have the potency to form cells of almost any type, but they differ in their path of formation. Blastemas on the other hand undergo lineage restricted differentiation, with many cells retaining some sort of memory of their origin (Kragl et al., 2009). iPSC formation is clearly a case of a dedifferentiation process. In contrast, it is likely that callus is not a dedifferentiated tissue; rather it forms by the activation of pre‐existing adult stem cells. Further, the source of the callus is normally only a few specific adult stem cells, unlike iPSCs which can be made from a whole range of mature cells from all three germ layers, mesoderm, ectoderm, and endoderm. While both iPSC and blastema formation involve dedifferentiation, blastema formation is more similar to callus formation since it has also been shown to occur at least partially through transdifferentiation, examples being retinal and tail regeneration in newts (Stewart & Stankunas, 2012). While it can be argued that a blastema occurs only in vivo, a recent system in which human mesenchymal cells were induced to form three‐dimensional condensate to mimic a blastema seems even more analogous to a plant callus formed in vitro. In this system, too, the cells have moderate levels of pluripotency factors but higher levels of early developmental stage markers (meso‐endoderm), indicating that they do not revert completely back to the ground state and can differentiate in vivo into a variety of cell types (Pennock et al., 2015).

## 6 DE NOVO ASSEMBLY OF REGULATORY INTERACTIONS, RECONSTITUTION OF SHOOT STEM CELLS, AND DE NOVO ORGANOGENESIS

Following the formation of pluripotent callus, a subsequent hormonal treatment is required to accomplish de novo organ regeneration (Fig. 2). Culturing the callus on cytokinin‐rich shoot inducing medium (SIM) favors shoot regeneration, whereas auxin‐rich root inducing medium (RIM) promotes root regeneration (Che et al., 2006; Gordon et al., 2007). Root regeneration from the callus is a relatively simple process as callus displays a root‐like trait. However, de novo shoot regeneration from callus is a more complex process.

**Figure 3:**
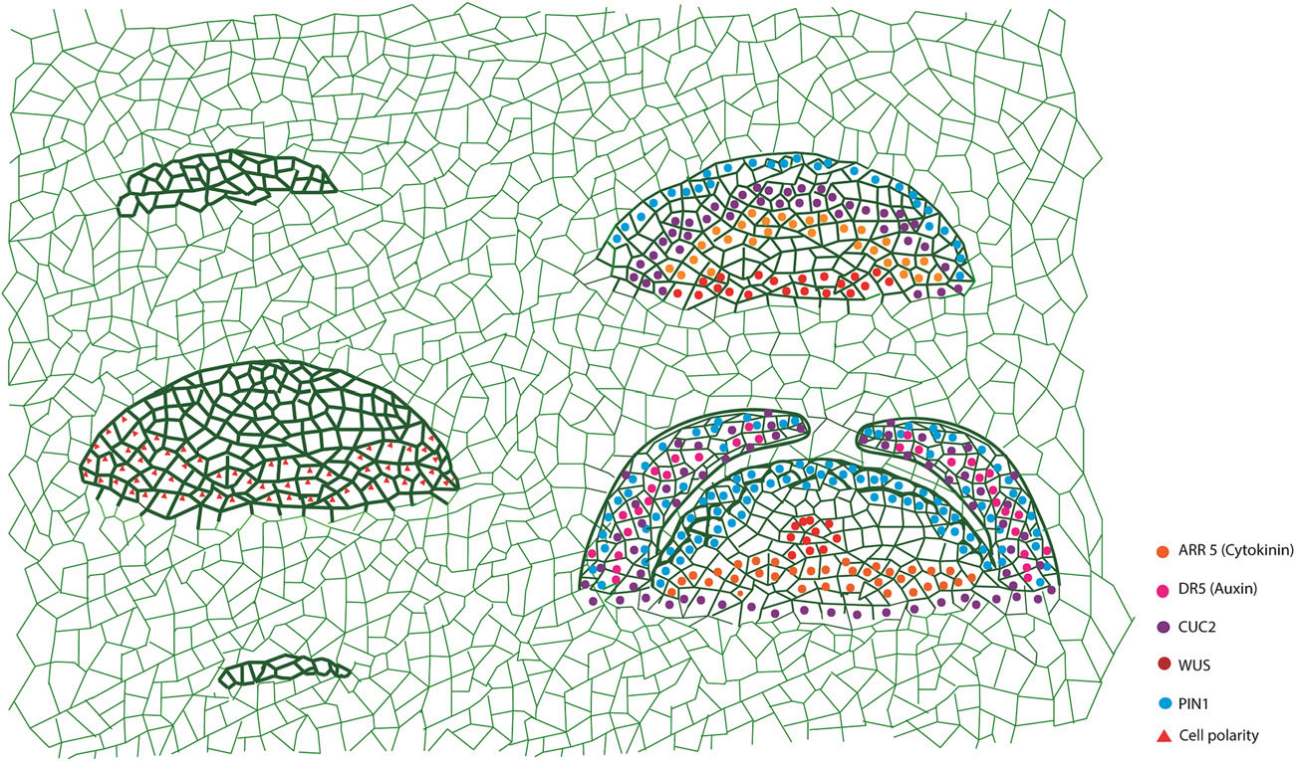
Schematic representing regeneration of shoot progenitors and assembly of regulatory interactions in the callus. Dome shaped structures on the left mark regenerating shoot progenitors at different developmental stages. The structure on the top right denotes shoot promeristem and the one on the bottom right denotes functional shoot meristem with organ primordia

During de novo shoot formation, culturing on SIM instigates the callus to create spatial domains of hormone perception that lead to partitioning of cell identity and re‐specification of cell fate from root‐like fate to shoot fate. In concurrence with the assembly of regulatory molecular interactions, these cells acquire the competence to regenerate shoot progenitors. Subsequently, formation of shoot promeristem and functional shoot apical meristem (SAM) with lateral organ patterning ensues (Gordon et al., 2007; Kareem et al., 2015) (Fig. 3). Morphologically, this process involves the greening of callus (also denoted as regenerating green foci) followed by the initiation of shoot. These sequential developmental events during de novo organogenesis are discussed in more detail in the following sections.

### 6.1 Uncoupling intermediate developmental phases of shoot regeneration: competence to regenerate shoot progenitors and completion of shoot formation

De novo shoot organogenesis progresses through several intermediate developmental phases. Formation of any of these phases such as green callus, shoot progenitor, or shoot promeristem is not an assurance of complete shoot regeneration. Studies on various *Arabidopsis* accessions identified several such instances of naturally occurring recalcitrance where regeneration is blocked at intermediate developmental phases, suggesting that the process of shoot regeneration can be separated into at least several steps (Motte et al., 2014). This study shows that there is a lack of significant correlation between callus formation or greening or primordia formation and shoot regeneration. The quantitative trait locus *RECEPTOR‐LIKE PROTEIN KINASE 1* (*RPK1*) regulates the variation in shoot regeneration capacity of different accessions. Consistent with this earlier study, the recent study on PLT mediated shoot regeneration demonstrates that the acquisition of competence to regenerate shoot progenitors can be uncoupled from the completion of shoot formation (Kareem et al., 2015). PLT3, PLT5, and PLT7 collectively regulate root stem cell maintenance regulators like PLT1 and PLT2 to make callus competent to regenerate shoot progenitors. The non‐competent *plt3;plt5;plt7* mutant callus displays deregulated expression of shoot‐specific genes, polar auxin transporter, and altered auxin response. The reconstitution of *PLT1* or *PLT2* in its endogenous spatio‐temporal domain in *plt3;plt5;plt7* callus reinstates the wild‐type callus trait and favors the regeneration of shoot progenitors which are marked with polar auxin transporter PINFORMED 1 (PIN1). But the green callus having shoot progenitors cannot proceed towards the completion of organ formation unless and until the shoot promoting factor like CUP SHAPED COTYLEDON 1 (CUC1) or CUC2 is activated. However, the frequency of shoot regeneration upon the activation of CUC is lower, suggesting that additional shoot promoting factors are required to achieve the optimum levels of complete shoot formation. Further, the independent activation of root stem cell maintenance regulators or shoot promoting factors alone cannot accomplish shoot regeneration in *plt3;plt5;plt7*. This suggests a two‐step mechanism of shoot regeneration in which PLT3, PLT5, and PLT7 regulate root stem cell maintenance regulators (PLT1 and PLT2) to establish pluripotency and thus the competence to regenerate shoot progenitors and further regulate shoot promoting factors (CUC) for the completion of shoot regeneration. This two‐step mechanism of shoot regeneration operates in all tissues irrespective of their origin. In parallel, de novo organogenesis from iPSCs in animals also follows multiple steps. Regeneration of organs such as kidney (Yokote, Yamanaka, & Yokoo, 2012) and intestine (Spence et al., 2011) from iPSCs progresses through intermediate developmental phases when it is exposed to the stepwise application of inductive cues.

### 6.2 De novo assembly of multiple regulatory interactions

**Figure 4:**
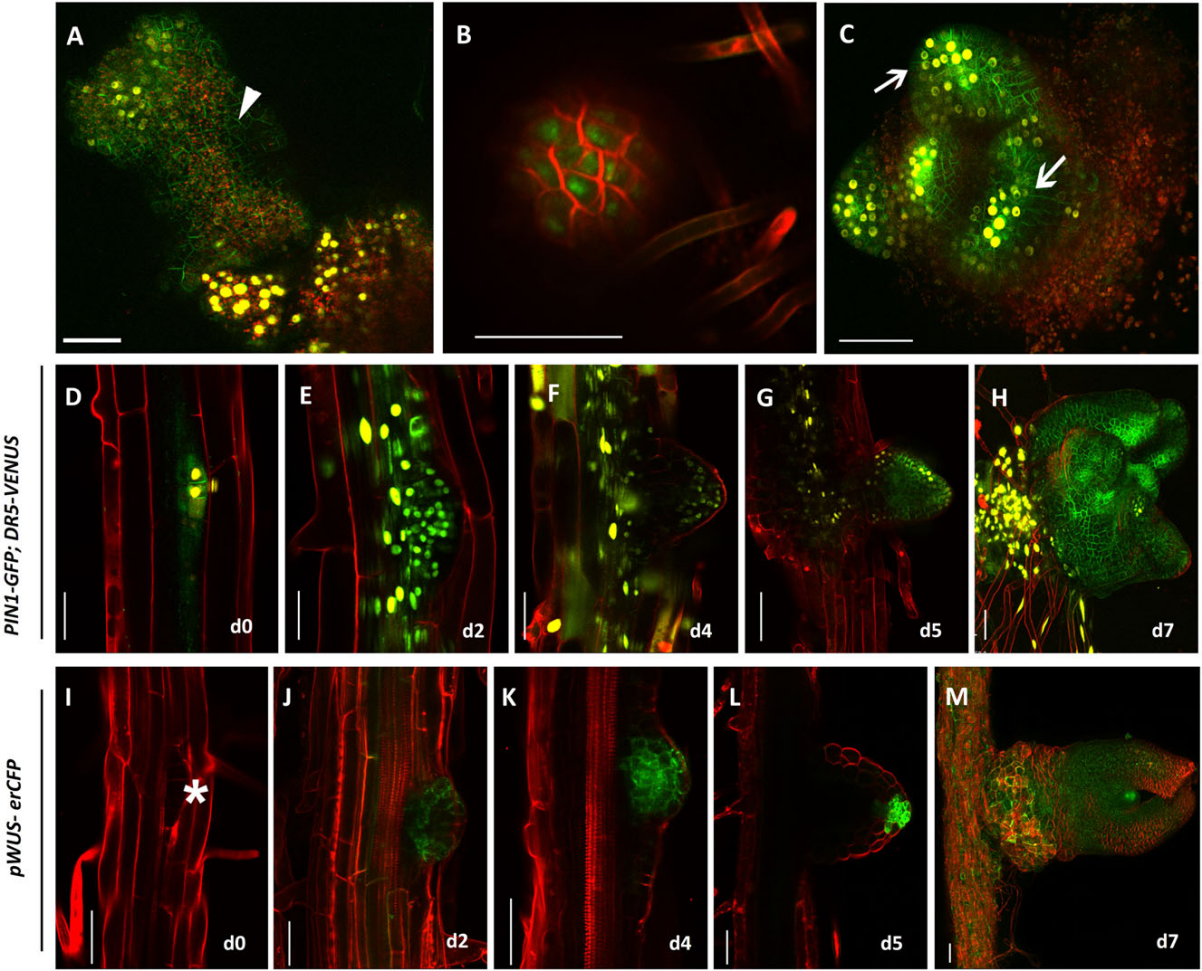
Intermediate developmental phases of de novo shoot regeneration. (A) Initial shoot progenitor cells are labeled with membrane localized PIN1‐GFP (green). Non‐progenitor cells are marked with DR5‐VENUS (yellow). (B) Developing shoot promeristem showing the confined expression of pWUS‐CFP (green). (C) Functional SAM bearing developing organ primordia marked with PIN1‐GFP and DR5‐VENUS. (D)−(H) Dynamic expression pattern of PIN1‐GFP and DR5‐VENUS during LRP to shoot conversion. (I)−(M) Expression pattern of pWUS‐erCFP during the various stages of LRP to shoot conversion. The scale bar represents 50 μm. (Images are reprinted from Kareem et al. (2015) with permission from Cell Press. License number: 3884940031971.)

The most critical part during de novo shoot regeneration is the positioning of shoot progenitors on the callus surface. Unlike embryonic SAM development, the positional information required for shoot progenitor formation followed by functional SAM development is not predetermined in de novo shoot regeneration. The positional information is absolutely governed by hormonal signaling mediated regulatory interactions during regeneration (Fig. 3). These hormonal signals activate certain key regulators of shoot regeneration. Various studies have shown that the key shoot stem cell regulator *WUSCHEL* (*WUS*) is one of the earliest shoot determinants induced in cytokinin‐rich medium (Gordon et al., 2007; Chatfield et al., 2013; Kareem et al., 2015, 2016). Cytokinin induces *WU*S through the activation of cytokinin receptors *ARABIDOPSIS HISTIDINE KINASE 2* (*AHK2*) and *AHK4* (Gordon, Chickarmane, Ohno, & Meyerowitz, 2009). Not only does *WUS* get activated by cytokinin, it can in turn induce cytokinin responses by directly suppressing the negative regulators of cytokinin signaling such as type‐A *ARRs* (Leibfried et al., 2005; Buechel et al., 2009). Thus cytokinin and *WUS* act in a positive feedback loop. In addition, ectopic overexpression of *WUS* is sufficient to make de novo shoots from callus on cytokinin‐free medium (Kareem et al., 2015). In association with WUS activation on cytokinin‐rich SIM, CUC2 gets restricted into a small population of rapidly dividing cells (Gordon et al., 2007). These rapidly dividing cells are the future shoot progenitor cells. Initially, WUS is observed to be expressed in the periphery of CUC2‐expressing cells and thus both of these regulatory molecules act in mutually exclusive functional domains. These non‐overlapping functional domains of WUS and CUC2 create a partition in callus for hormonal response and cellular identity. Similar to the *CUC2* expression pattern, *PLT3*, *PLT5*, and *PLT7* genes are also restricted to rapidly dividing shoot forming cells (Kareem et al., 2015). It has been shown that *WUS* expression is epigenetically regulated by both DNA methylation and histone modifications during shoot regeneration (Li et al., 2011). It is possible that epigenetic regulators might contribute to the spatial distribution pattern of WUS and help to position the shoot stem cell niche in callus. Together with *CUC2* and other unknown regulators, *WUS* facilitates the formation of shoot progenitors. The shoot progenitors are marked with both PIN1 and CUC2 while WUS labels the cells surrounding the progenitors (Fig. 4A). At this moment a dynamic shift in the expression of *WUS* follows. It gets confined to the center of dome shaped shoot progenitors and further leads to the formation of shoot promeristem (Fig. 4B). The *CUC2* expression pattern is also changed and it is visualized in the peripheral zone of shoot promeristem in a radial pattern (Gordon et al., 2007).

### 6.3 Organization of functional shoot meristem and completion of shoot formation

Subsequent to the formation of shoot promeristem, polar localization of PIN1 marks the future anlagen of organ initiation. Concomitant with polarized PIN1 upregulation, the key regulator of shoot meristem maintenance *SHOOT MERISTEMLESS* (*STM*) appears in the promeristem (Gordon et al., 2007). *STM* prevents the meristematic cells from differentiating into lateral organs. Simultaneously several other regulators including auxin signaling molecule *MONOPTEROS* (MP) are upregulated in shoot promeristem (Ckurshumova, Smirnova, Marcos, Zayed, & Berleth, 2014). Also, the stem cells residing at the center of shoot meristem express *CLAVATA3* (*CLV3*) (Gordon et al., 2007). A functional SAM is thus established (Fig. 4C). The subsequent developmental events for the patterning of lateral organs are a recapitulation of in planta shoot development.

Several other factors have also been implicated in shoot regeneration such as *ENHANCER OF SHOOT REGENERATION 1* (*ESR1*) and *ESR2* which trigger shoot regeneration in a hormone‐independent medium (Banno, Ikeda, Niu, & Chua, 2001; Ikeda, Banno, Niu, Howell, & Chua, 2006). *ESR2* activates *CUC1* at the transcript level to promote shoot regeneration (Ikeda et al., 2006). The roles of *CUC1* and *CUC2* to promote shoot regeneration have been demonstrated (Daimon, Takabe, & Tasaka, 2003; Hibara, Takada, & Tasaka, 2003) and *CUC* genes are activated by PLT3, PLT5, and PLT7 (Kareem et al., 2015). Various regulators of de novo shoot regeneration such as homeobox containing proteins, KNOX proteins, double AP2 containing proteins, and ARFs are conserved across the plant species. Thus the regulatory actions of these genes are likely to be conserved. Despite the growing knowledge about the key regulators, regulatory interactions among several of these are yet to be established to generate the larger regulatory network involved in de novo shoot regeneration.

### 6.4 Whether or not to remember embryonic developmental phases

A beautiful work by Efroni et al. (2016) has very recently shown that, upon severe damage, tissue repair follows an embryo‐like sequence to reconstitute stem cells in regenerating root. Similarly, during de novo shoot regeneration, the shoot stem cell niche is reconstituted in the pluripotent callus. A careful follow‐up of the temporal sequence of gene expression in live imaging of a regenerating callus demonstrates the progressive assembly of the spatial expression of a battery of genes that eventually culminate in the formation of a confined shoot stem cell niche (Gordon et al., 2007). During very early embryogenesis, key shoot stem cell regulators confine their expression to a few cells at the tip of the apical pole of the embryo (Radoeva & Weijers, 2014). CUC gets localized to the periphery of these cells. In strong *wus* mutant, the embryonic SAM cannot be traced. But, post‐embryonically, shoot meristem displays “on” and “off” activity in *wus* mutant (Laux, Mayer, Berger, & Jurgens, 1996; Mayer et al., 1998). Mutants like *wus* and *stm* make de novo shoots albeit at a lower frequency (Barton & Poethig, 1993; Gordon et al., 2007). Based on existing studies on the spatio‐temporal expression pattern of shoot‐specific genes and their mutant phenotype during embryogenesis and de novo shoot regeneration, it is difficult to infer that reconstitution of a shoot stem cell niche during de novo shoot stem cell formation follows an embryo‐like sequence. These differences could be attributed to a different mode of regeneration, that is, tissue repair upon disruption of multicellularity versus assembly of the entire plant system from a regenerative mass.

The future discovery of additional shoot‐specific regulators and the real‐time live imaging of the dynamic cellular events during embryogenesis and de novo shoot regeneration might reveal if there is any cellular process during de novo shoot regeneration that follows an embryo‐like sequence.

### 6.5 De novo regeneration and somatic embryogenesis: two routes to the same destination

In addition to de novo organogenesis, in vitro regeneration can also be accomplished by somatic embryogenesis (Zimmerman, 1993; Pulianmackal et al., 2014). Although both systems are capable of generating complete plantlets, these methods can be distinguished from each other. Somatic embryos are formed by the treatment of somatic cells with high concentration of auxin such as 2,4‐D (Ikeda‐Iwai, Satoh, & Kamada, 2002; Su et al., 2009). While somatic embryogenesis involves the formation of bipolar structures bearing shoot and root meristem, de novo organogenesis completely bypasses embryonic developmental phases to produce monopolar structures of multicellular origin bearing either root or shoot identity (Thorpe & Stasolla, 2001). Furthermore, somatic embryos do not maintain any vascular connection with the explant tissue, unlike the shoot or root formed as a result of de novo organogenesis. Interestingly, a high level of basic helix−loop−helix transcription factor bHLH109 induces somatic embryogenesis while its low level is associated with de novo shoot organogenesis (Nowak & Gaj, 2016). Since during somatic embryogenesis root and shoot pole is pre‐specified, it is not dependent on the assembly of regulatory interactions for positional information. In contrast, reconstitution of stem cells and assembly of spatio‐temporally controlled regulatory interactions are central to positional information during de novo organogenesis.

## 7 DIRECT REPROGRAMMING

Besides callus mediated regeneration, direct reprogramming of root to shoot and shoot to root occurs in nature as well as under in vitro conditions. Although the root and shoot of plants arise from distinct embryonic regions, they can still regenerate organs different from their own origin, such as root from shoot and vice versa. This can largely be attributed to common evolutionary origins of root and shoot. It is believed that roots of modern day plants evolved from the shoots of primitive plants (Gifford & Foster, 1989; Friedman, Moore, & Purugganan, 2004). Therefore, these two structures are likely to retain the potential to get reprogrammed to the other fate. Not surprisingly, plants such as ferns, many monocots, dicots such as curry leaves, guava, and some tree species possess the natural capacity to generate shoot from root as a part of vegetative propagation (Wittrock, 1884; Holm, 1925). Such reprogramming potential has been widely exploited in horticulture. For instance, a variety of plant parts such as shoot cuttings, root cuttings, and even leaf cuttings have been used for plant propagation. Stem cuttings have been used for propagating common garden plants like chrysanthemum, carnation, and rose (Stangler, 1956) while plants such as raspberry and horseradish can be multiplied by root cuttings (Dore, 1953). Leaves of *Bryophyllum*, *Saintpaulia*, and *Sansevieria* are capable of forming entire plantlets bearing root and shoot (Goethe, 1820; Broertjes, Haccius, & Weidlich, 1968; Henson & Wareing, 1977).

Direct conversion of root to shoot and vice versa under in vitro culture conditions can further stretch the limits of plant propagation to produce a larger number of plantlets in the minimum possible time. An added advantage of in vitro culture compared to vegetative propagation is that even the plant parts which are not conventionally considered as vegetative propagules can be used as explants for multiplication under appropriate hormone treatment. Unlike indirect regeneration, direct regeneration bypasses an intervening callus phase thereby helping to avoid or reduce somaclonal variation associated with prolonged callus culture (Kareem et al., 2016). Moreover, the direct regeneration process can serve as a general model to study the mechanism underlying transdifferentiation (conversion of one cell type into another). Transdifferentiation is also reported in animal systems wherein a differentiated cell can be directly converted into a different lineage without passing through an intermediate pluripotent state by the forced expression of tissue‐specific transcription factors (Ieda et al., 2010; Vierbuchen et al., 2010; Sekiya & Suzuki, 2011).

### 7.1 Root to shoot

An ideal example of direct reprogramming is the induction of shoot from root. Culturing of root explants on cytokinin‐rich medium promotes the direct conversion of LRP into shoot (Atta et al., 2009; Chatfield et al., 2013; Kareem et al., 2015, 2016). Real‐time monitoring of dynamic cellular events using live imaging enables us to understand the early molecular events during LRP to shoot conversion (Fig. 4D−M). High cytokinin induces the expression of shoot‐specific genes such as *WUS* in LRP which can lead to reprogramming of root cells to shoot fate. During the early stages of cell fate transition, cells pass through a transient developmental phase in which both root‐specific and shoot‐specific genes are expressed (Kareem et al., 2016). Subsequently, there is an elevation in the expression of shoot‐specific genes and disappearance of root‐specific genes. The interactions of multiple shoot‐specific regulatory molecules establish the shoot meristem identity in regenerating structure followed by organ initiation (Chatfield et al., 2013; Kareem et al., 2016). However, this conversion process is highly influenced by a number of factors such as stage of the LRP, ecotype of the plant, cytokinin concentration, and external conditions such as light and temperature (Kareem et al., 2016).

### 7.2 Shoot to root

Similar to the conversion of root to shoot, direct conversion of shoot to root is also possible under in vitro culture conditions. This approach is of significance when not all propagules used in vegetative propagation undergo rooting as is the case during hardening of plants in micropropagation. Therefore understanding the factors that affect root formation from the shoot explant can help to overcome the recalcitrance to form root in the plant species of interest. Root formation from the shoot explants requires external auxin supplements. Auxin initiates the reprogramming of procambium or pericycle‐like cells of shoot explant into root fate (Greenwood, Cui, & Xu, 2001; Ahkami et al., 2009; Da Rocha Correa, Troleis, Mastroberti, Mariath, & Fett‐Neto, 2012; De Almeida, De Almeida, Graner, Brondani, & De Abreu‐Tarazi, 2012). In addition to auxin a number of other phytohormones such as ethylene, giberrelin, ABA, and extrinsic culture parameters such as nutrition, light, temperature, and the ecotype of the explants are also important during root organogenesis (Coleman, Huxter, Reid, & Thorpe, 1980; Verstraeten, Beeckman, & Geelen, 2013; Su & Zhang, 2014; Welander et al., 2014). Interestingly, studies have shown that rooting can be induced from detached leaf without external auxin treatment (Liu et al., 2014). Upon wounding, *YUCCA* (*YUC*) gene mediated auxin biosynthesis occurs at the site of injury which is essential for the de novo root regeneration (Chen, Tong et al., 2016). The WUSCHEL‐RELATED HOMEOBOX 11 (WOX11) and WOX12 transcription factors respond to this auxin maximum by upregulating LBD16 and LBD29 resulting in a switch from procambium and nearby parenchyma cell fate to root founder cell fate (Liu et al., 2014). LBDs may also be involved in the consequent transition to root primordia. So the wound induced adventitious root formation in the absence of external hormone application highlights that the default pathway adopted is of root fate. Wounding induces the NAC pathway that is independent of the auxin maxima mediated cell fate switching (Chen, Cheng et al., 2016). Therefore, similar to wound induced callus formation and subsequent regeneration, the wound response pathway can also initiate direct root organogenesis.

In addition to the external inductive cues, cell fate transition can also occur by the ectopic activation of intrinsic genetic factors. For instance, inducible ectopic overexpression *WUS* can trigger shoot from root on hormone‐free medium (Gallois, Nora, Mizukami, & Sablowski, 2004; Kareem et al., 2015). Similarly ectopic induction of *PLT2* can activate root formation from shoot apex (Galinha et al., 2007). Taken together, it can be comprehended that direct shoot and root organogenesis are under the control of extrinsic as well as intrinsic factors. The molecular dissection of massive cellular reprogramming during cell fate transition can be instrumental in understanding the mechanism underlying transdifferentiation. Furthermore, this knowledge can be used to enhance the direct regeneration capacity of recalcitrant species by adopting a combinatorial approach of standardization.

## 8 PERSPECTIVE

Most organisms are born with the gift of regeneration that has been tested time and again, over the course of evolution. However, the extent of this capability varies, perhaps to suit the adversities posed by their natural habitats. While some organisms like axolotl can regenerate a lost body part like the Deadpool superhero, plants are much ahead of the animal kingdom in their regeneration capability. Provided that the ideal inductive cues are given, plants have the unique ability to be reborn from a handful of cells or even a single somatic cell (Vasil & Vasil, 1980) which houses the blueprint and the formula necessary to recreate a complete plant. In a very recent study, an externally imposed electric field influences plant regeneration in addition to other external inductive cues like hormones and injury (Kral, Ougolnikova, & Sena, 2016). The underlying molecular basis is yet to be revealed.

Despite the distinctions, it is interesting to note the convergence in the mechanisms underlying regeneration in both plant and animal kingdoms. A common challenge that both the systems face is the maintenance of the spatio‐temporal balance between self‐renewal and differentiation. While the finer details are different, a common theme emerges that master transcription factors (genetic control) are necessary and sufficient to recreate this process in both plants and animals (Gallois et al., 2004; Takahashi & Yamanaka, 2006; Smith et al., 2013; Kareem et al., 2015). However, epigenetic regulation is critical for fine‐tuning this process and has a major role to play in vivo as well (Lafos et al., 2011; Li et al., 2011; Lee et al., 2014). There have been instances of epigenetic regulation of transcription factors in both plants and animals (Farthing et al., 2008; Luo et al., 2012). However, the reverse is poorly understood in the plant system compared to animals (Liang & Zhang, 2013). Studies may uncover similar homologous mechanisms in the plant system (Weiste & Droge‐Laser, 2014; De Lucas et al., 2016). This crosstalk between genetic and epigenetic regulators occurs in a bi‐directional manner. The signaling pathways acting in concert with transcription factors can control cell fate decisions via epigenetic modifications such as DNA methylation, and histone acetylation and methylation (Anzola et al., 2010; Fagnocchi, Mazzoleni, & Zippo, 2016). The interplay between genetic and epigenetic factors may be responsible for making cells containing the same genetic information differentiate into diverse kinds of cells with varying levels of potency. However, among the two modes of regulation, epigenetics may be more easily influenced by a mere modification of extrinsic culture conditions (Moussaieff et al., 2015) compared to modulating the genetic regulators.

**Figure 5:**
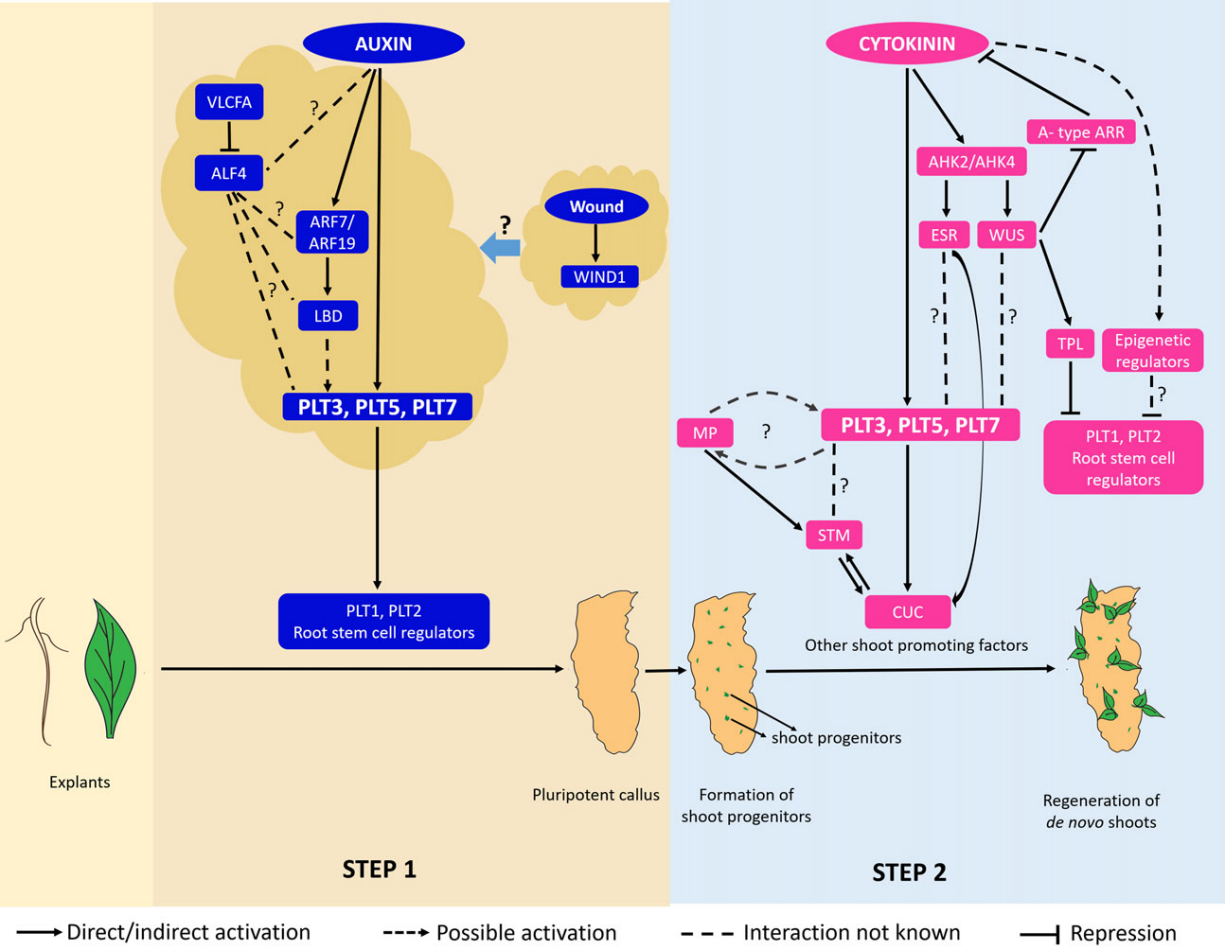
Larger regulatory network involved in two‐step mechanism of shoot regeneration. A detailed description of the regulatory interactions is given in the text. Auxin requires the activity of ALF4 to induce the callus (Sugimoto et al., 2010). WUS activates the expression of *TOPLESS* (*TPL*) (Busch et al., 2010) and TPL represses *PLT1* and *PLT2* (Smith & Long, 2010 during embryogenesis). MP (Ckurshumova et al., 2014) and CUC (Daimon et al., 2003) induce *STM* expression during regeneration. STM in turn activate *CUC* during in‐planta shoot development (Spinelli, Martin, Viola, Gonzalez & Palatnik, 2011).

These studies, however, have only scratched the tip of the iceberg. There is a long way to go before we acquire a better understanding of the galaxy of genetic and epigenetic players involved in regeneration. The next step then would be to unravel the mechanism by which the crosstalk occurs between these regulators. Although plants have been hailed for their higher regeneration potential, we are yet to nail down all the factors responsible for this cellular plasticity. In addition, although the signature factors of embryonic, root, and shoot development and de novo regeneration have been elucidated, the larger regulatory network involving these players is still being described (Fig. 5). It is interesting to note that similar or the same set of factors in different contexts can regulate distinct modes of regeneration. Ectopic overexpression of *WUS* or *PLT5* can induce de novo shoot regeneration (Gallois et al., 2004; Kareem et al., 2015) and somatic embryogenesis (Zuo, Niu, Frugis, & Chua, 2002; Tsuwamoto, Yokoi, & Takahata, 2010; Siligato et al., 2016). Similarly, *ALF4* plays significant role in callus formation (Sugimoto et al., 2010) as well as wound healing during grafting (Melnyk et al. 2015). Furthermore, a careful analysis at high cellular resolution and an interpretation of regulatory interactions during de novo regeneration are required to identify whether these reflect embryonic or post‐embryonic developmental programs in a scenario where organisms utilize the very same set of regulators in two different developmental contexts.

In the long run, the lessons learnt in regeneration from the dynamic array of species can be applied to enhance the regeneration capacity of the recalcitrant species which are of relevance in agricultural sciences and forestry. Besides the huge application in green culture industries for food plants, de novo regeneration offers a beautiful system to address a number of fundamental questions pertaining to acquisition of the pluripotent state, reconstitution of stem cells, and the dynamics of assembly of regulatory interactions leading to the culmination of de novo organogenesis. Furthermore, plants can serve as a general model to study the conserved nature of the factors, which would also pave the way for the discovery of similar regulatory interactions in other kingdoms which may have a direct impact on human welfare. Human civilization began in close connection with simple domestication of plants and animals. In today's era, it has become important to understand the biological processes similar among these organisms to meet the demands of an ever expanding population and the challenges of disease and degeneration.
